# Peer Review of “Representing Physician Suicide Claims as Nanopublications: Proof-of-Concept Study Creating Claim Networks”

**DOI:** 10.2196/39860

**Published:** 2022-07-01

**Keywords:** physician suicide, suicide, suicide prevention, physician well-being, physician mental health, nanopublication, physician, doctor, mental health, semantic publishing, bibliometrics, claim network, information distortion, misinformation

*This is a peer-review report submitted for the paper “Representing Physician Suicide Claims as Nanopublications: Proof-of-Concept Study Creating Claim Networks.”*

## Round 1 Review

### General Comments

This paper [1] tries to address a myth that has been and continues to be perpetuated around the number of US physician suicides. It tries to put forward an approach to addressing myths in a way to better inform specific populations (ie, in this case, physicians) of the reality of suicide in the profession.

### Specific Comments

#### Major Comments

1. This is an important effort and should be applauded.

2. This confirms that the myth remains in existence, despite minor changes to it, and should be either verified or dispelled.

3. The approach presented to address situations as described is one that has merit and can be useful to many where there is interest and desire. The paper was well written, clear, data driven, and of a good length. I would have liked a bit more in the discussion section (eg, implications could be stronger) and a simple conclusion statement at the end.
